# Statistical analysis of an RNA titration series evaluates microarray precision and sensitivity on a whole-array basis

**DOI:** 10.1186/1471-2105-7-511

**Published:** 2006-11-22

**Authors:** Andrew J Holloway, Alicia Oshlack, Dileepa S Diyagama, David DL Bowtell, Gordon K Smyth

**Affiliations:** 1 Ian Potter Foundation Centre for Cancer Genomics and Predictive Medicine, Peter MacCallum Cancer Centre, St Andrew's Place, East Melbourne, Victoria 3002, Australia; 2 Walter and Eliza Hall Institute, 1G Royal Parade, Parkville, Victoria 3050, Australia

## Abstract

**Background:**

Concerns are often raised about the accuracy of microarray technologies and the degree of cross-platform agreement, but there are yet no methods which can unambiguously evaluate precision and sensitivity for these technologies on a whole-array basis.

**Results:**

A methodology is described for evaluating the precision and sensitivity of whole-genome gene expression technologies such as microarrays. The method consists of an easy-to-construct titration series of RNA samples and an associated statistical analysis using non-linear regression. The method evaluates the precision and responsiveness of each microarray platform on a whole-array basis, i.e., using all the probes, without the need to match probes across platforms. An experiment is conducted to assess and compare four widely used microarray platforms. All four platforms are shown to have satisfactory precision but the commercial platforms are superior for resolving differential expression for genes at lower expression levels. The effective precision of the two-color platforms is improved by allowing for probe-specific dye-effects in the statistical model. The methodology is used to compare three data extraction algorithms for the Affymetrix platforms, demonstrating poor performance for the commonly used proprietary algorithm relative to the other algorithms. For probes which can be matched across platforms, the cross-platform variability is decomposed into within-platform and between-platform components, showing that platform disagreement is almost entirely systematic rather than due to measurement variability.

**Conclusion:**

The results demonstrate good precision and sensitivity for all the platforms, but highlight the need for improved probe annotation. They quantify the extent to which cross-platform measures can be expected to be less accurate than within-platform comparisons for predicting disease progression or outcome.

## Background

In recent years there has been a rapidly growing understanding of how gene expression reflects and determines biological states. This has come about through the widespread use of microarray expression profiling [1]. Yet there have been concerns about the accuracy and reproducibility of the technology. Some early studies reported poor reproducibility and dramatic differences between platforms [2-5]. Although other studies have generally reported better accuracy and agreement [6-8], especially later studies using more developed statistical methods [9-12], the early concern has contributed to an explosion in the number of publications comparing microarray platforms or assessing microarray accuracy.

Despite the growing number of publications, only a limited number of methods are available to assess the accuracy of genome-scale expression platforms. Before discussing the strengths and weakness of the various strategies, it is necessary to dissect exactly what is meant by accuracy. There are several dimensions to platform accuracy. The first major dimension is consistency, i.e., the ability of the platform to agree with itself. This can be further divided into reproducibility or precision on one hand, and dynamic range or sensitivity on the other. These features determine the ability of the platform to distinguish differentially expressed transcripts from those which are not. The sensitivity can be further examined to check whether the measured probe intensities increase linearly with transcript expression level. This determines the ability of the platform to return accurate estimates of the fold changes for differentially expression genes. The second major dimension is probe annotation. Accurate annotation determines the ability of the platform to agree with independent measures of differential expression for the same genes. It might be for example that a microarray platform accurately measures the expression level for some gene, but the probe is incorrectly annotated as another gene. Another possibility is that two microarray platforms might both accurately measure expression for the correct gene, but might nevertheless disagree because they respond to different splice-variants or isoforms of that gene [9,13-16]. Annotation accuracy is likely to improve for all platforms as knowledge of the genome improves. We can view self-consistency as the innate accuracy of the platform because it can be improved only by a change in the underlying technology.

Many platform comparison articles use variability between technical replicates to measure precision [4,6,10,15-22], but this doesn't measure sensitivity or linearity. To measure sensitivity, it is necessary to introduce genes which are known to be differentially expressed. PCR is the traditional method for validating microarray discoveries, so some studies use quantitative PCR to determine the true differential expression status for a subset of genes [3,7,9,10,13,23-26]. This approach is practical only for a small proportion of the probes, and has some other disadvantages which are discussed below. Another method of introducing known fold changes is to spike-in a small number of artificial genes into the RNA sample at known concentrations [17,27-29]. This technique requires that alien control probes be printed onto the arrays and the corresponding transcripts spiked into the RNA samples before labeling and hybridization. Spike-in controls however are inevitably platform specific and hence are more suited to comparing different processing methods for a given platform than for comparing different platforms. Even for a given platform, spike-in controls suffer from a number of shortcomings. Only a small number of distinct transcripts can be spiked-in and these are often at a higher message abundance than the endogenous genes and at relatively large fold changes. Finally, the fact that the spike-in RNA is not extracted with the main RNA sample and has to be added separately means that the spike-in probes will not follow the same intensity-dependent normalization curve as the regular probes. This means that the spike-in probes will generally need to be normalized separately to the other probes and will not necessarily be representative of the precision of the other probes.

Most platform comparison studies begin by matching probes between platforms, and then measuring the agreement between platforms for the common genes [4,6-10,12,15,16,19-22,25,30-33]. Although natural, this approach suffers from a number of problems. Firstly, there is no perfect or unambiguous strategy for matching probes. Matching on UniGene ID is clearly insufficient because each platform may have multiple probes with possibly different expression profiles mapped to the same ID. The same problem applies to other gene classification systems. More detailed sequence analysis may attempt to position the probes at particular loci in the genome [24,30], but even so an unambiguous matching of probes between platforms is highly unlikely. When platforms disagree, one cannot easily identify the source of disagreement, whether this is due to innate imprecision of the platforms or incorrect annotation. More basically, when two platforms disagree the comparison does not tell us which platform is correct, or even if either is correct. Even the validation of microarray results by PCR can be subject to these problems, especially if the splice variants for the gene in question are not completely described.

A small number of studies have used a dilution series of RNA samples to construct samples with a range of fold changes [27,28,32,34]. Two other studies have taken the approach mixing mRNA from two sources in known quantities for the same purpose [16,35].

In this article we use a series of mixed mRNA samples and introduce a more systematic method of analysis. Mixtures of mRNA were derived from two cell lines in varying proportions to cover a large dynamic range in gene expression. A statistical methodology is introduced for evaluating the precision, sensitivity and linearity of microarray platforms. The novel design allows the series of arrays to be analyzed as a whole. The mixture series give predictable changes in fold change against which the actual pattern of observed log-ratios can be compared. There are several advantages to this design. Firstly, each microarray platform is evaluated on a whole-array basis. Every probe on the arrays adds information in assessing the precision of the platform as each probe will behave according to the mixtures series. Secondly, there is no need to match probes across platforms as each platform yields its own measure. On the other hand, if the additional step of matching probes across platforms is performed, then our method provides a decomposition of variability into within- and between-platform components for each gene. This provides, for the first time, a quantitative assessment of the degree of systematic disagreement between the platforms. Our method could be applied to evaluate the precision of any whole-genome gene expression technology, not only microarrays.

We use our methodology to assess and compare four widely used microarray platforms: two commercial platforms, Affymetrix and Agilent, and two academically produced platforms, spotted cDNA and oligonucleotide arrays. This selection includes three two-color platforms and one single-channel platform (Affymetrix). Our comparison includes careful consideration of special issues which affect the different technologies including dye-effects for the two-color platforms and experimental design differences between the two-color and single-channel platforms.

Our intention is to evaluate the best-case scenario for each platform rather than to evaluate laboratory to laboratory variation as several other studies have done [10,29,31,36]. Hence we hybridized all the two-color microarrays in a single experienced laboratory.

Of equal importance as the cross-platform issues is the evaluation of alternative data processing algorithms for a given platform. Pre-processing algorithms by which expression measures are quantified from microarray images include background correction, normalization and summarization methods [37-39]. There is a slowly emerging appreciation in the literature of the size of the noise reduction that can be achieved by state-of-the-art pre-processing methods [16,27-29,32,34,40,41]. For this study we used what we consider to be best-practice pre-processing methods for each platform. Our data and associated statistical analysis provide an objective means to compare different pre-processing methods for each platform. To illustrate this we compare several popular methods of pre-processing Affymetrix GeneChip expression values and show that the method used in our study performs better than the alternatives.

## Results

### The titration series design

**Figure 1:**
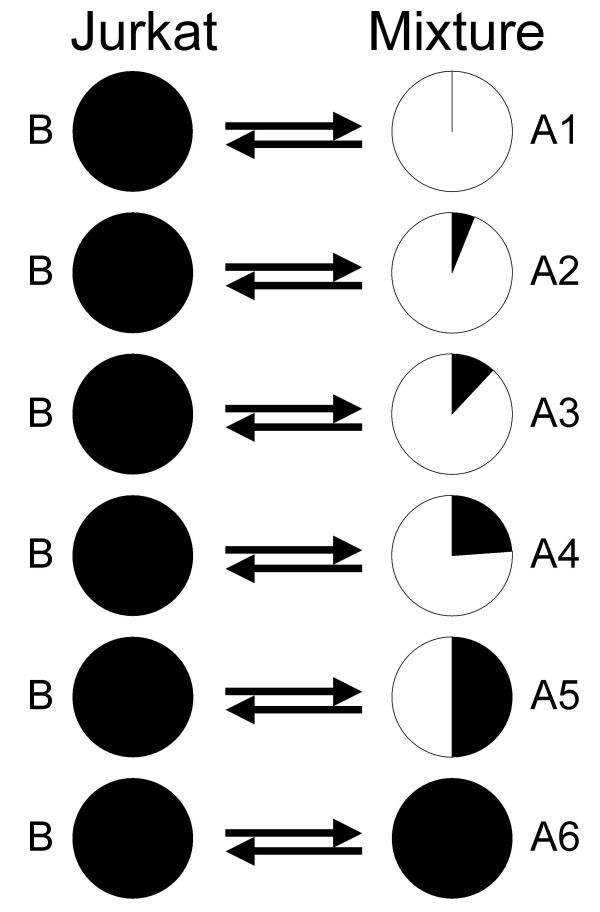
**Design of experiment**. This schematic represents the 12 microarrays which were hybridized for each of the two-color platforms. Each arrow represents one array, with the head pointing to the sample labeled Cy5 (red) and the tail at the sample labeled Cy3 (green). The labels A1 to A6 and B correspond to Table 1, where the exact mixing proportions are given.

Six RNA mixes were prepared ranging from pure MCF7 (A1) through four mixtures of MCF7 and Jurkat (A2–A5) to pure Jurkat (A6) (Table 1). For each of the three two-color platforms, twelve arrays were hybridized (Figure 1). Each of the six RNA mixes was compared to pure Jurkat (B), with a dye-swap pair of arrays used for each comparison. For the single channel platform, fourteen arrays were hybridized, two for each of RNA mixes A1–A6 and B. The rationale for this design is that the comparison of pure RNAs A1 vs B should show considerable differential expression and can be used to evaluate the responsiveness or sensitivity of the platforms, while the same vs same comparison A6 vs B should show no differential expression and can be used to evaluate the variability or specificity of the platforms. The graduated mixes A2–A5 can be used to evaluate the ability of each platform to faithfully track fold changes as the relative expression changes. Our analysis is in two major parts. First we examine the precision, i.e., the repeatability, of each platform individually. This analysis uses all non-control probes on the arrays. This analysis does not attempt to match probes between platforms but simply assumes each platform contains a comparable selection of probes. Later we do match probes across the four platforms, to the extent that is possible, and examine the concordance of the four platforms on these common genes.

**Table 1:** Mixing proportions used for titration series

**Sample**	**MCF7 c**	**Jurkat 1-c**
**A1**	100%	0%
**A2**	94%	6%
**A3**	88%	12%
**A4**	76%	24%
**A5**	50%	50%
**A6**	0%	100%
**B**	0%	100%

Samples A1 through A6 were created by mixing total mRNA from MCF7 breast epithelial cells with mRNA derived from the Jurkat T cell line in the proportions shown. The sample B was used as a reference RNA in all two-color hybridizations (spotted cDNA, spotted oligonucleotide and Agilent). For Affymetrix hybridizations, samples A1 through B were hybridized independently.

**Figure 2:**
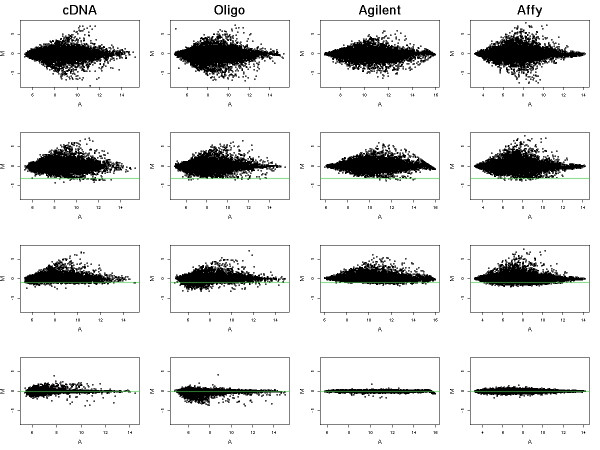
**Selected MA plots for the titration series**. M (log_2 _transformed fold change value) was plotted versus A (total intensity for that spot) for all genes on the arrays. Columns represent Spotted cDNA, Spotted oligonucleotide, Agilent and Affymetrix. The top row shows the A1 sample (MCF7) vs Jurkat, the second row shows the A3 mixture (88% MCF7, 12% Jurkat) vs Jurkat, the third row shows A5 (50% MCF7, 50% Jurkat) vs Jurkat and the bottom row is A6 (100% Jurkat) in a self-self hybridization. As expected the A1 sample shows the most differential expression in the series of arrays. Similarly, the A6 sample shows very little differential expression. The green line represents the theoretical minimum fold change. No elements should report a fold change below this line.

Figure 2 shows a representative selection of MA-plots for arrays in the experiment. The rows of the plot correspond to the RNA mixes A1, A3, A5 and A6 respectively and the four columns to the four platforms. For the two-color platforms, each MA-plot displays the relationship between the log-ratios (M-values) and log-intensities (A-values) for one array [42]. For the single-channel platform, each MA-plot is constructed by comparing all the log-intensities (E-values) of one array to the average log-intensities of the two B arrays [43]. The top row corresponds to the pure MCF7 vs Jurkat comparison (A1 vs B), the second row to 88% MCF7 vs Jurkat (A3 vs B), the third row to 50% MCF7 vs Jurkat (A5 vs B) and the bottom row is the Jurkat vs Jurkat comparison (A6 vs B). The plots are orientated so that positive M-values correspond to higher expression in MCF7 and negative M-values correspond to higher expression in Jurkat. An ideal microarray platform would show substantial differential expression on the top row and successively decreasing differential expression going down the figure. The MA-plots in the bottom row should ideally show horizontal lines at M = 0 with no vertical variation at all. All four microarray platforms show this qualitative behavior with considerable spread of M-values above and below the zero line in the top row and very little variation at the bottom of the figure. The M-values for the Agilent and Affymetrix platforms are particularly tight around M = 0 in the bottom plots while the spotted arrays show somewhat more variation from M = 0. Looking more closely at the Agilent and Affymetrix columns, all the Affymetrix plots show more vertical variation than the corresponding Agilent plots, suggesting that the Affymetrix platform is more responsive than the Agilent, returning larger fold changes, but is also more noisy.

### Nonlinear regressions

For each probe *p*, let *F*_*p *_be the intensity corresponding to the expression level of the corresponding transcript in MCF7 and let *J*_*p *_be the intensity corresponding to its expression in Jurkat. If *c *is the proportion of MCF7 in a particular RNA mix, and if the observed intensity is proportional to expression, then the intensity of the mix should be *cF*_*p *_+ (1 - *c*)*J*_*p*_. Also let *R*_*p *_= *F*_*p*_/*J*_*p *_be the expression ratio (fold change) between MCF7 and Jurkat for that probe. The fold change between the RNA mix and the Jurkat reference should be {*cF*_*p *_+ (1 - *c*)*J*_*p*_}/*J*_*p *_= *cR*_*p *_+ 1 - *c*. This shows that, although we do not know the true values of *F*_*p *_or *J*_*p *_for any gene, the log-expression and log-ratio values must change in a predictable way across the titration series.

For the single-channel platform, let *E*_*pj *_be the normalized log-intensity for probe *p *and array *j*, *j *= 1,...,14. The theoretical log-intensity for probe *p *and array *j *is

*α*_*pj *_= log_2_{*c*_*j*_*F*_*p *_+ (1 - *c*_*j*_)*J*_*p*_}

where *c*_*j *_is the proportion of MCF7 in the RNA mix used for array *j*. The observed log-intensity *E*_*pj *_can be represented as

*E*_*pj *_= log_2_(Mixture) = *α*_*pj *_+ *ε*_*pj *_    (1)

where *ε*_*pj *_represents measurement error. We assume that the *ε*_*pj *_are independent with mean zero and with probe dependent standard deviations *ϕ*_*p*_. For the two-color platforms, let *M*_*pi *_be the normalized log-ratio for probe *p *on array *i*, *i *= 1,...,12. The theoretical log-ratio for probe *p *and array *i *is

*β*_*pi *_= log_2_(*c*_*i*_*R*_*p *_+ 1 - *c*_*i*_)

where *c*_*i *_is the proportion of MCF7 in RNA mix used for array *i*. The observed log-ratio can be represented as

*M*_*pi *_= log_2_(Mixture/Jurkatt) = *d*_*i*_*β*_*pi *_+ *ε*_*pi *_    (2)

where *ε*_*pi *_represents measurement error and *d*_*i *_= -1 if the array is dye-swapped and 1 otherwise. We assume that the *ε*_*pi *_are independent with mean zero and with probe dependent standard derivations *σ*_*p*_.

Note that the minimum value that the fold change *R*_*p *_can take is zero, for a probe whose transcript is present in Jurkat but completely absent in MCF7. The minimum possible value for the log-ratio *β*_*pi *_is therefore log_2_(1 - *c*_*i*_), the log of the proportion of Jurkat in the mix. The titration series introduces in this way a theoretical lower bound on the range of possible log-ratios. The horizontal green lines in Figure 2 represent this bound. Any M-value which falls below this line does so in error. In general, all four platforms maintain M-values consistently above the line, with the greatest transgressions occurring for the spotted oligonucleotide arrays.

**Figure 3:**
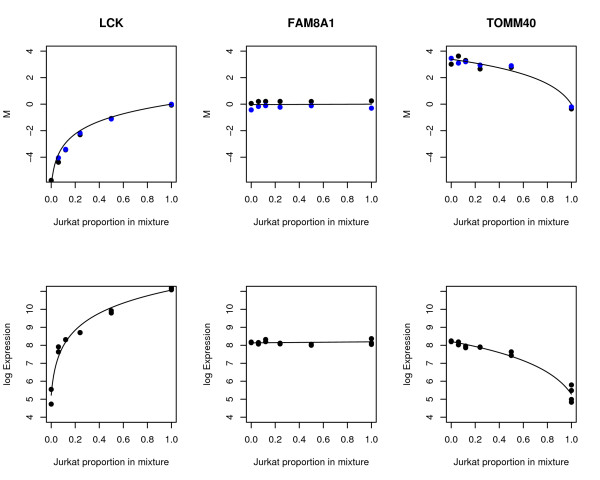
**Fitted nonlinear curves for three example genes**. The curves show the tend-lines predicted by the titration series of mixed RNA samples. The top three plots show curves fitted to the cDNA M-values, which are indicated as dots. Black dots are M-values, blue dots are dye-swap M-values which have been reversed in sign for the plot. The bottom three plots show curves fitted to the Affymetrix log-intensities, shown as dots.

The standard deviations *σ*_*p *_and *φ*_*p *_are the keys to quantifying the precision of the platforms, because they represent the array-to-array variability which is expected in measuring log-ratios or log-expression values. Our approach is to view equations (1) and (2) as nonlinear regression equations for the *E*_*pj *_and the *M*_*pi*_, with unknown regression parameters *F*_*p *_and *J*_*p *_for equation (1) and *R*_*p *_for equation (2). These equations are fitted by least squares to the available data for each probe to obtain estimated fold changes and precisions for that probe. Regression equation (1) was fitted to the 14 *E*_*pj *_data values for each Affymetrix probe-set and equation (2) was fitted to the 12 *M*_*pi *_data values for each probe on each two-color platform. A complete worked example of the computations for one gene is given in the Supplementary Materials [44]. Examples of fitted curves are shown in Figure 3. In each case, the fitted curve is that which minimizes the sum of squared deviations of the data values from the curve. These calculations yielded an estimated fold change *R*_*p *_for each probe on each two-color platform, which we call the *consensus estimate *of the fold change between MCF7 and Jurkat for each probe, because it is derived from the entire series of twelve arrays. The consensus estimator is more precise than the simple mean log-ratio that could be obtained from the pure MCF7 and Jurkat arrays alone. The residual standard error *s*_*p *_from each regression is our estimate for *σ*_*p*_. Note that sp2
 MathType@MTEF@5@5@+=feaafiart1ev1aaatCvAUfKttLearuWrP9MDH5MBPbIqV92AaeXatLxBI9gBaebbnrfifHhDYfgasaacH8akY=wiFfYdH8Gipec8Eeeu0xXdbba9frFj0=OqFfea0dXdd9vqai=hGuQ8kuc9pgc9s8qqaq=dirpe0xb9q8qiLsFr0=vr0=vr0dc8meaabaqaciaacaGaaeqabaqabeGadaaakeaacqWGZbWCdaqhaaWcbaGaemiCaahabaGaeGOmaidaaaaa@30A3@ is a consistent and approximately unbiased estimator for σp2
 MathType@MTEF@5@5@+=feaafiart1ev1aaatCvAUfKttLearuWrP9MDH5MBPbIqV92AaeXatLxBI9gBaebbnrfifHhDYfgasaacH8akY=wiFfYdH8Gipec8Eeeu0xXdbba9frFj0=OqFfea0dXdd9vqai=hGuQ8kuc9pgc9s8qqaq=dirpe0xb9q8qiLsFr0=vr0=vr0dc8meaabaqaciaacaGaaeqabaqabeGadaaakeaaiiGacqWFdpWCdaqhaaWcbaGaemiCaahabaGaeGOmaidaaaaa@30FE@ by standard nonlinear least squares theory [45]. For each Affymetrix probe-set, the intensities *F*_*p *_and *J*_*p *_were estimated from the observed log-expression values by non-linear least squares. This produced consensus estimates of MCF7 and Jurkat expression for each probe from the entire series fourteen arrays. The residual standard error from each regression is our estimate of *φ*_*p*_. In this way, precision estimates were obtained for each probe on each platform. Note that we applied least squares to the *E*_*pj *_rather than to the un-logged expression values and to the *M*_*pi *_rather than the fold changes because the logged values are more nearly symmetrically and normally distributed.

### Dye effects

Another consideration with the two-color platforms is the possibility of probe-specific dye bias, i.e., the tendency of particular probes to incorporate one of the dyes more readily than the other [46]. Dye-swaps were incorporated into our design specifically to detect and balance such effects. Equation (2) can be modified to allow for a dye-effect as

**Figure 4:**
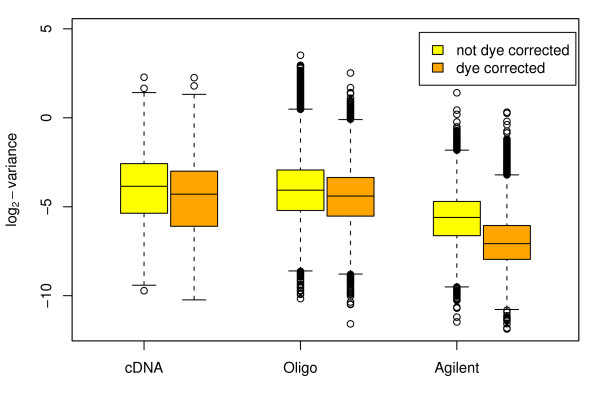
**Boxplots of genewise variances for the two-color platforms before and after removing the gene-specific dye-effects**. The orange boxplots give variances after correction for the gene-specific dye-effect and show that the variances are noticeably reduced. Lower values correspond to greater precisions. The improvement is most marked for the Agilent platform. Each unit on the vertical axis corresponds to doubling of statistical information.

*M*_*pi *_= *δ*_*p *_+ *d*_*i*_*β*_*pi *_+ *ε*_*pi *_    (3)

where the intercept term *δ*_*p *_represents the probe specific dye effect. If probe *p *does show a specific dye bias, then including the intercept term will reduce the estimate of *σ*_*p *_as the new equation (3) more closely models the behavior of the probe. If not, the estimated *σ*_*p *_will on average remain the same, although the residual degrees of freedom for the regression will decrease by one. Boxplots of estimated σp2
 MathType@MTEF@5@5@+=feaafiart1ev1aaatCvAUfKttLearuWrP9MDH5MBPbIqV92AaeXatLxBI9gBaebbnrfifHhDYfgasaacH8akY=wiFfYdH8Gipec8Eeeu0xXdbba9frFj0=OqFfea0dXdd9vqai=hGuQ8kuc9pgc9s8qqaq=dirpe0xb9q8qiLsFr0=vr0=vr0dc8meaabaqaciaacaGaaeqabaqabeGadaaakeaaiiGacqWFdpWCdaqhaaWcbaGaemiCaahabaGaeGOmaidaaaaa@30FE@ with and without the dye effect term are given in Figure 4. It is clear that accounting for dye effects improved the fits for all three platforms. For the Agilent arrays the improvement is particularly noticeable, nearly halving the median estimated σp2
 MathType@MTEF@5@5@+=feaafiart1ev1aaatCvAUfKttLearuWrP9MDH5MBPbIqV92AaeXatLxBI9gBaebbnrfifHhDYfgasaacH8akY=wiFfYdH8Gipec8Eeeu0xXdbba9frFj0=OqFfea0dXdd9vqai=hGuQ8kuc9pgc9s8qqaq=dirpe0xb9q8qiLsFr0=vr0=vr0dc8meaabaqaciaacaGaaeqabaqabeGadaaakeaaiiGacqWFdpWCdaqhaaWcbaGaemiCaahabaGaeGOmaidaaaaa@30FE@. Each of the two-color platforms was hybridized with identical material from a large pool of labeled mRNA, so the larger effect for Agilent arrays is not due to the labeled material applied to the arrays. We conclude that all two color platforms show small but noticeable dye effects for a large proportion of the probes. These dye effects can be estimated and removed in a suitably designed experiment allowing inference to be conducted with improved precision and less bias.

### Precision of individual platforms

Comparing estimated *σ*_*p *_values shows that the Agilent platform is more precise, i.e., more repeatable, than the two spotted platforms, which are very similar in precision. The median *σ*_*p *_s are 0.226, 0.218 and 0.0861 for the cDNA, oligo and Agilent platforms respectively, after correction for dye-effects. These standard deviations correspond to plus/minus fold changes of 17%, 16% and 6% respectively. The step from the spotted platforms to Agilent is substantial. The numbers suggest that (0.218/0.0861)^2 ^= 6.4 technical replicate oligo arrays are required to estimate a typical fold change with the same precision as one Agilent array.

Comparing the two-color platforms with Affymetrix is not so straightforward, because *σ*_*p *_measures the precision of log-ratios while *φ*_*p *_measures the precision of log-intensities. Quantitative comparison can only be done in the context of specific experimental designs. If two Affymetrix arrays are used to estimate log-fold changes between two RNA targets, the standard deviation of the estimate for probe *p *should be 2
 MathType@MTEF@5@5@+=feaafiart1ev1aaatCvAUfKttLearuWrP9MDH5MBPbIqV92AaeXatLxBI9gBaebbnrfifHhDYfgasaacH8akY=wiFfYdH8Gipec8Eeeu0xXdbba9frFj0=OqFfea0dXdd9vqai=hGuQ8kuc9pgc9s8qqaq=dirpe0xb9q8qiLsFr0=vr0=vr0dc8meaabaqaciaacaGaaeqabaqabeGadaaakeaadaGcaaqaaiabikdaYaWcbeaaaaa@2DB9@*φ*_*p*_, assuming the two arrays to be independent and to have equal variances. With two-color arrays, we need to distinguish between direct comparisons, in which the two RNA targets of interest are hybridized competitively to the same array, and indirect comparisons, whereby one channel of each array is hybridized with a common reference sample [47]. If two two-color arrays are used to compare two RNA targets indirectly via a common reference, the standard deviation of the estimated log-fold change for probe *p *should be 2
 MathType@MTEF@5@5@+=feaafiart1ev1aaatCvAUfKttLearuWrP9MDH5MBPbIqV92AaeXatLxBI9gBaebbnrfifHhDYfgasaacH8akY=wiFfYdH8Gipec8Eeeu0xXdbba9frFj0=OqFfea0dXdd9vqai=hGuQ8kuc9pgc9s8qqaq=dirpe0xb9q8qiLsFr0=vr0=vr0dc8meaabaqaciaacaGaaeqabaqabeGadaaakeaadaGcaaqaaiabikdaYaWcbeaaaaa@2DB9@*σ*_*p*_, again assuming the arrays to be independent with equal variances. On the other hand, if RNA targets are compared directly on the same arrays, the standard deviation would be *σ*_*p *_using one array and *σ*_*p*_/2
 MathType@MTEF@5@5@+=feaafiart1ev1aaatCvAUfKttLearuWrP9MDH5MBPbIqV92AaeXatLxBI9gBaebbnrfifHhDYfgasaacH8akY=wiFfYdH8Gipec8Eeeu0xXdbba9frFj0=OqFfea0dXdd9vqai=hGuQ8kuc9pgc9s8qqaq=dirpe0xb9q8qiLsFr0=vr0=vr0dc8meaabaqaciaacaGaaeqabaqabeGadaaakeaadaGcaaqaaiabikdaYaWcbeaaaaa@2DB9@ using two replicate arrays [48]. Table 2 gives the precisions, i.e., standard deviations, with which a log-fold change would be estimated from a minimal experiment for each of the platforms. For the two-color platforms, results are given for both a direct comparison using one array and for an indirect comparison using two arrays. For Affymetrix, the results assume a comparison between two arrays. Agilent is the most precise platform, providing that the dye-effect has been adjusted as indicated above. The spotted arrays are nearly as precise as Affymetrix if they use direct comparison but much less precise if they do not.

**Table 2:** Standard deviation with which a typical log-fold change is measured using a minimal experiment.

		**Std Deviation**		
**Design**	**Platform**	**Q1**	**Median**	**Q3**

**Direct**	**cDNA**	.121	.226	.353
	**Oligo**	.147	.218	.313
	**Agilent**	.063	.086	.122
**Indirect**	**cDNA**	.171	.319	.500
	**Oligo**	.208	.308	.442
	**Agilent**	.090	.122	.173
	**Affymetrix**	.131	.167	.211

The table gives the median standard deviation across probes together with the first and third quartiles. For the two-color platforms the design may be direct using one array or indirect via a common reference using two arrays. For Affymetrix, two arrays are used. The two-color calculations assume that estimates of probe-specific dye effects are available, .e.g., from a larger experiment including dye-swaps.

**Figure 5:**
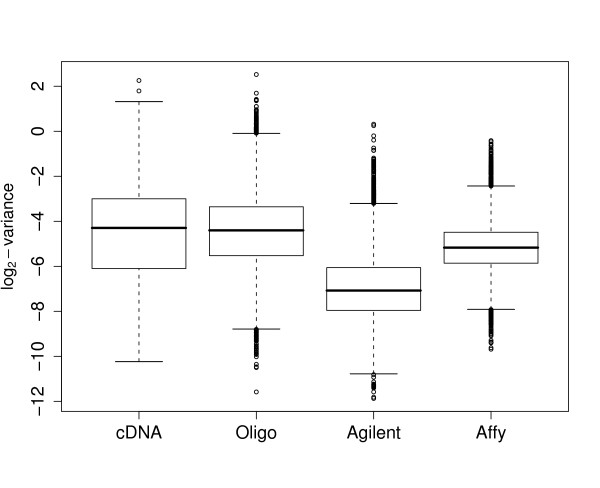
**Boxplots of genewise standard deviations with which a log-fold change is measured using the smallest possible experiment for each platform**. Lower values correspond to greater precision and each unit of the vertical axis corresponds to a doubling of statistical information. For the two-color platforms, values of log_2_σ^p2
 MathType@MTEF@5@5@+=feaafiart1ev1aaatCvAUfKttLearuWrP9MDH5MBPbIqV92AaeXatLxBI9gBaebbnrfifHhDYfgasaacH8akY=wiFfYdH8Gipec8Eeeu0xXdbba9frFj0=OqFfea0dXdd9vqai=hGuQ8kuc9pgc9s8qqaq=dirpe0xb9q8qiLsFr0=vr0=vr0dc8meaabaqaciaacaGaaeqabaqabeGadaaakeaaiiGacuWFdpWCgaqcamaaDaaaleaacqWGWbaCaeaacqaIYaGmaaaaaa@310E@ are plotted, corresponding to a direct comparison with one array after adjustment for probe-specific dye-effects. For Affymetrix, values of log_2_2ϕ^p
 MathType@MTEF@5@5@+=feaafiart1ev1aaatCvAUfKttLearuWrP9MDH5MBPbIqV92AaeXatLxBI9gBaebbnrfifHhDYfgasaacH8akY=wiFfYdH8Gipec8Eeeu0xXdbba9frFj0=OqFfea0dXdd9vqai=hGuQ8kuc9pgc9s8qqaq=dirpe0xb9q8qiLsFr0=vr0=vr0dc8meaabaqaciaacaGaaeqabaqabeGadaaakeaacqaIYaGmiiGacuWFvpGAgaqcamaaBaaaleaacqWGWbaCaeqaaaaa@3116@ are plotted corresponding to a comparison of two arrays. The median lines correspond to the direct design median values in Table 2, and the boxplot outlines correspond to the Q1 and Q3 given in Table 2.

Boxplots of the probe-wise standard deviations are shown in Figure 5. For the two-color platforms the results assume a direct design and are corrected for probe-specific dye effects, so the situation is the same as for the top half of Table 2. The conclusions are the same as for Table 2. The Agilent arrays perform clearly the best, followed by Affymetrix. For all the platforms there is a considerable range of precisions across the probes. The full distribution of probe-wise standard deviations for each platform is shown as a density histogram in Supplementary Figure 1[44].

**Figure 6:**
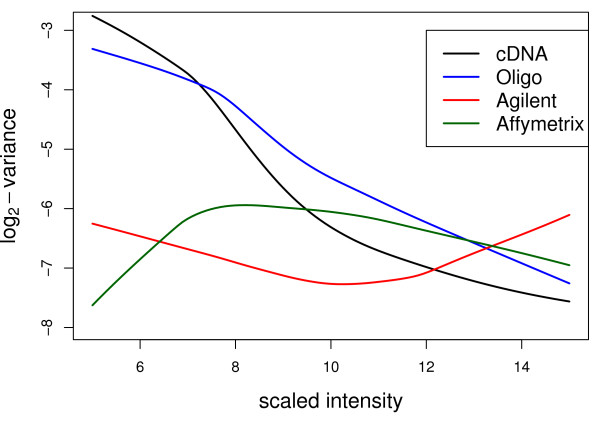
**Estimated precisions for different platforms as a function of expression level**. The curves are lowess trend curves fitted to the log_2_σp2
 MathType@MTEF@5@5@+=feaafiart1ev1aaatCvAUfKttLearuWrP9MDH5MBPbIqV92AaeXatLxBI9gBaebbnrfifHhDYfgasaacH8akY=wiFfYdH8Gipec8Eeeu0xXdbba9frFj0=OqFfea0dXdd9vqai=hGuQ8kuc9pgc9s8qqaq=dirpe0xb9q8qiLsFr0=vr0=vr0dc8meaabaqaciaacaGaaeqabaqabeGadaaakeaaiiGacqWFdpWCdaqhaaWcbaGaemiCaahabaGaeGOmaidaaaaa@30FE@ values for the two-color platforms, corresponding to a direct comparison with one array with dye-effect adjustment, and to the log_*p *_2ϕp2
 MathType@MTEF@5@5@+=feaafiart1ev1aaatCvAUfKttLearuWrP9MDH5MBPbIqV92AaeXatLxBI9gBaebbnrfifHhDYfgasaacH8akY=wiFfYdH8Gipec8Eeeu0xXdbba9frFj0=OqFfea0dXdd9vqai=hGuQ8kuc9pgc9s8qqaq=dirpe0xb9q8qiLsFr0=vr0=vr0dc8meaabaqaciaacaGaaeqabaqabeGadaaakeaaiiGacqWFvpGAdaqhaaWcbaGaemiCaahabaGaeGOmaidaaaaa@3107@ values for Affymetrix, corresponding to a comparison of two arrays. The horizontal axis is the average log-intensity over the A1 arrays for the two color platforms and over the A1 and B arrays for Affymetrix. Each unit on the vertical axis corresponds to a doubling of statistical information.

It is well known that the variability of expression measures can depend on the abundance of the gene transcript, although the pre-processing methods we used are designed in part to reduce this dependence. Figure 6 displays trend lines of σp2
 MathType@MTEF@5@5@+=feaafiart1ev1aaatCvAUfKttLearuWrP9MDH5MBPbIqV92AaeXatLxBI9gBaebbnrfifHhDYfgasaacH8akY=wiFfYdH8Gipec8Eeeu0xXdbba9frFj0=OqFfea0dXdd9vqai=hGuQ8kuc9pgc9s8qqaq=dirpe0xb9q8qiLsFr0=vr0=vr0dc8meaabaqaciaacaGaaeqabaqabeGadaaakeaaiiGacqWFdpWCdaqhaaWcbaGaemiCaahabaGaeGOmaidaaaaa@30FE@ and 2ϕp2
 MathType@MTEF@5@5@+=feaafiart1ev1aaatCvAUfKttLearuWrP9MDH5MBPbIqV92AaeXatLxBI9gBaebbnrfifHhDYfgasaacH8akY=wiFfYdH8Gipec8Eeeu0xXdbba9frFj0=OqFfea0dXdd9vqai=hGuQ8kuc9pgc9s8qqaq=dirpe0xb9q8qiLsFr0=vr0=vr0dc8meaabaqaciaacaGaaeqabaqabeGadaaakeaaiiGacqWFvpGAdaqhaaWcbaGaemiCaahabaGaeGOmaidaaaaa@3107@ against mean A-value, and these show generally decreasing trends of variability versus intensity. The spotted arrays are far more variable for lower intensity probes while the commercial platforms maintain precision more evenly over the entire intensity range. Agilent appears to have greater precision than Affymetrix for probes in the middle range of intensities. The precisions of the four platforms tend to converge for the highest intensity probes. In Figure 6, the A-values for the different platforms have been scaled to have the same range.

### Pure error and bias

It should be kept in mind when interpreting *σ*_*p *_and *φ*_*p *_that they are overall measures of residual variation which respond to both the variability between technical replicate arrays (pure error) and any failure of the nonlinear regression curves to track the correct fold changes (bias). Although the regression curves give a visually good fit to the empirical data (Figure 3), there may nevertheless be evidence for lack of fit. Such lack of fit might arise from a failure of the microarray spot intensities to respond to expression levels with perfect proportionality, or possibly from small pipetting errors in preparing the titration series. The pure error and bias components are additive according to *σ*^2 ^= σPure Error2
 MathType@MTEF@5@5@+=feaafiart1ev1aaatCvAUfKttLearuWrP9MDH5MBPbIqV92AaeXatLxBI9gBaebbnrfifHhDYfgasaacH8akY=wiFfYdH8Gipec8Eeeu0xXdbba9frFj0=OqFfea0dXdd9vqai=hGuQ8kuc9pgc9s8qqaq=dirpe0xb9q8qiLsFr0=vr0=vr0dc8meaabaqaciaacaGaaeqabaqabeGadaaakeaaiiGacqWFdpWCdaqhaaWcbaGaeeiuaaLaeeyDauNaeeOCaiNaeeyzauMaeeiiaaIaeeyrauKaeeOCaiNaeeOCaiNaee4Ba8MaeeOCaihabaGaeGOmaidaaaaa@3C67@ + (Bias)^2 ^for each probe. We estimated the pure error and bias components by decomposing the residual sum of squares from each of the nonlinear regressions (1) and (3) into pure error and lack of fit components, and formed F-statistics to test for lack of fit by dividing the lack of fit mean square by the pure error mean square [49] (Section 3.4). The F-statistics were found to be randomly distributed around unity (data not shown), which is evidence that there is little or no systematic lack of fit. None of the F-statistics were statistically significant after Bonferroni or Holm adjustment for multiple testing across probes. As an extra precaution, in case lack of fit exists but is not detected by the statistical tests because of lack of power, we estimated the size of the lack of fit variance component for each probe. For all probes which show differentially expression between MCF7 and Jurkat, the estimated lack of fit variance component (squared bias) was either zero or extremely small compared with the sums of squares due to the nonlinear regression trend (data not shown). We conclude that the nonlinear regressions describe the data more than adequately for our purposes and that pure error is the dominant component of the residual standard errors.

### Concordance between platforms

We now turn to the question of systematic discordance between the platforms, and for this purpose we need to match probes corresponding to common genes across the platforms. UniGene IDs were obtained for all probes, using the UniGene build of 10 November 2003, and 3,636 genes were found to be present on all four platforms. This was not sufficient to match probes across platforms because most (56%) of the common genes were represented by more than one probe, or probe set in the case of Affymetrix, on at least one of the platforms. In order to have an objective method of matching probes uniquely across platforms, we chose for each gene the probe on each platform with the highest average expression level. In other words, we chose the probe whose transcript is most highly represented in the cell lines used in the study. Choosing a single representative probe on each platform in this way was preferable to averaging multiple probes for a given gene, for two reasons. Firstly, different probes for the same gene on the same platform in many cases showed quite different expression profiles, so that averaging would be inappropriate. Secondly, taking averages of different numbers of probes would prevent the platforms from being compared on the same basis.

**Figure 7:**
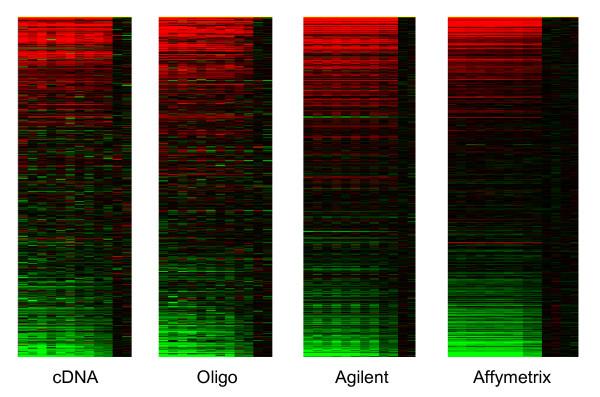
**Heat map representations of gene expression across the titration series for 3636 genes common to all platforms**. The horizontal axis corresponds to genes sorted from highest to lowest log-fold-change, according to an average of the four platforms for the A1 samples. The vertical axis corresponds to the 12 arrays (A1–A6 with dye-swaps) for the two-color platforms and to 14 arrays (A1–A6, B) for Affymetrix. Expression values are log-ratios: genes in red are Jurkat specific while genes in green are MCF7 specific. For Affymetrix, log-ratios are computed relative to the average of the Jurkat (A6, B) samples.

**Figure 8:**
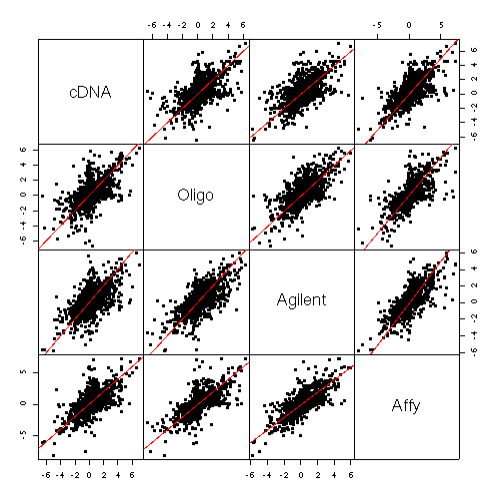
**Scatterplots of log-fold-change (log_2_*R*_*p*_) for the 3636 genes with probes on all four platforms**. The plot shows broad agreement but also much disagreement between the four platforms.

The probes matched in this way showed broadly similar behavior across the platforms but also noticeable differences (Figure 7). Genes are sorted by average MCF7 vs Jurkat log-ratio across platforms and columns correspond to individual arrays progressing from A1 on the left to A6 on the right. Dye-swapped arrays are un-swapped here *in silico*, i.e., are shown with M-values reversed in sign. As expected, we observe a progressive reduction in the number of differentially expressed genes from left to right, although with somewhat more variability for the two spotted platforms. The dye-effect noted above can be seen in the alternating vertical stripes for the two-color platforms, most noticeable for Agilent. The fold-change values *R*_*p *_estimated by the nonlinear regression are also in broad agreement across the platforms, although there is considerable scatter around equality (Figure 8). Points lying on the *y *= 0 line for each pane in Figure 8 indicate probes which fail to respond in that platform. All four platforms seem to be returning a similar range of fold changes. Cross-platforms correlations range from 0.6 between cDNA and oligo to 0.75 between Agilent and Affymetrix (Table 3). Affymetrix enjoys the best agreement with the other platforms and cDNA the least. The oligo platform is better correlated with Agilent and Affymetrix than the cDNA platform despite being no more precise, suggesting that the annotation of the Compugen probes is superior to that of the cDNA probes.

**Table 3:** Cross-platform correlations of log-fold-changes for 3636 genes with probes on all four platforms. Each correlation corresponds to a pane in Figure 8.

	**cDNA**	**Oligo**	**Agilent**	**Affy**
**cDNA**	1.000	0.600	0.621	0.668
**Oligo**	0.600	1.000	0.687	0.707
**Agilent**	0.621	0.687	1.000	0.746
**Affy**	0.668	0.707	0.746	1.000

By comparing the estimated fold changes with the platform precisions, the cross-platform variability can be decomposed into within-platform and between-platform components. That is, the disagreement between any two platforms can be separated into the variation inherent in the two platforms and the variation caused by trying to match probes across different technologies. Let *M*_1 _and *M*_2 _be log-ratios for a particular gene from two technologies, i.e., *M*_1 _is the log-ratio observed on an array of platform 1 and *M*_2 _is observed on an array of platform 2. For Affymetrix, the log-ratio would be obtained by comparing two arrays. The expected squared discrepancy between the two log-ratios is the sum of variance and squared-bias:

*E*{(*M*_1 _- *M*_2_)^2^} = Variance + Bias^2^

= σ12
 MathType@MTEF@5@5@+=feaafiart1ev1aaatCvAUfKttLearuWrP9MDH5MBPbIqV92AaeXatLxBI9gBaebbnrfifHhDYfgasaacH8akY=wiFfYdH8Gipec8Eeeu0xXdbba9frFj0=OqFfea0dXdd9vqai=hGuQ8kuc9pgc9s8qqaq=dirpe0xb9q8qiLsFr0=vr0=vr0dc8meaabaqaciaacaGaaeqabaqabeGadaaakeaaiiGacqWFdpWCdaqhaaWcbaGaeGymaedabaGaeGOmaidaaaaa@3085@ + σ22
 MathType@MTEF@5@5@+=feaafiart1ev1aaatCvAUfKttLearuWrP9MDH5MBPbIqV92AaeXatLxBI9gBaebbnrfifHhDYfgasaacH8akY=wiFfYdH8Gipec8Eeeu0xXdbba9frFj0=OqFfea0dXdd9vqai=hGuQ8kuc9pgc9s8qqaq=dirpe0xb9q8qiLsFr0=vr0=vr0dc8meaabaqaciaacaGaaeqabaqabeGadaaakeaaiiGacqWFdpWCdaqhaaWcbaGaeGOmaidabaGaeGOmaidaaaaa@3087@ + {log_2 _(*cR*_1 _+ 1 - *c*) - log_2_(*cR*_2 _+ 1 - *c*)}^2^

where σ12
 MathType@MTEF@5@5@+=feaafiart1ev1aaatCvAUfKttLearuWrP9MDH5MBPbIqV92AaeXatLxBI9gBaebbnrfifHhDYfgasaacH8akY=wiFfYdH8Gipec8Eeeu0xXdbba9frFj0=OqFfea0dXdd9vqai=hGuQ8kuc9pgc9s8qqaq=dirpe0xb9q8qiLsFr0=vr0=vr0dc8meaabaqaciaacaGaaeqabaqabeGadaaakeaaiiGacqWFdpWCdaqhaaWcbaGaeGymaedabaGaeGOmaidaaaaa@3085@ and σ22
 MathType@MTEF@5@5@+=feaafiart1ev1aaatCvAUfKttLearuWrP9MDH5MBPbIqV92AaeXatLxBI9gBaebbnrfifHhDYfgasaacH8akY=wiFfYdH8Gipec8Eeeu0xXdbba9frFj0=OqFfea0dXdd9vqai=hGuQ8kuc9pgc9s8qqaq=dirpe0xb9q8qiLsFr0=vr0=vr0dc8meaabaqaciaacaGaaeqabaqabeGadaaakeaaiiGacqWFdpWCdaqhaaWcbaGaeGOmaidabaGaeGOmaidaaaaa@3087@ are the precisions σp2
 MathType@MTEF@5@5@+=feaafiart1ev1aaatCvAUfKttLearuWrP9MDH5MBPbIqV92AaeXatLxBI9gBaebbnrfifHhDYfgasaacH8akY=wiFfYdH8Gipec8Eeeu0xXdbba9frFj0=OqFfea0dXdd9vqai=hGuQ8kuc9pgc9s8qqaq=dirpe0xb9q8qiLsFr0=vr0=vr0dc8meaabaqaciaacaGaaeqabaqabeGadaaakeaaiiGacqWFdpWCdaqhaaWcbaGaemiCaahabaGaeGOmaidaaaaa@30FE@ for this gene on the two platforms and *R*_1 _and *R*_2 _are the fold changes *R*_*p *_for this gene on two platforms. For the A1 samples the proportion of MCF7 is *c *= 1 so

*E*{(*M*_1 _- *M*_2_)^2^} = σ12
 MathType@MTEF@5@5@+=feaafiart1ev1aaatCvAUfKttLearuWrP9MDH5MBPbIqV92AaeXatLxBI9gBaebbnrfifHhDYfgasaacH8akY=wiFfYdH8Gipec8Eeeu0xXdbba9frFj0=OqFfea0dXdd9vqai=hGuQ8kuc9pgc9s8qqaq=dirpe0xb9q8qiLsFr0=vr0=vr0dc8meaabaqaciaacaGaaeqabaqabeGadaaakeaaiiGacqWFdpWCdaqhaaWcbaGaeGymaedabaGaeGOmaidaaaaa@3085@ + σ22
 MathType@MTEF@5@5@+=feaafiart1ev1aaatCvAUfKttLearuWrP9MDH5MBPbIqV92AaeXatLxBI9gBaebbnrfifHhDYfgasaacH8akY=wiFfYdH8Gipec8Eeeu0xXdbba9frFj0=OqFfea0dXdd9vqai=hGuQ8kuc9pgc9s8qqaq=dirpe0xb9q8qiLsFr0=vr0=vr0dc8meaabaqaciaacaGaaeqabaqabeGadaaakeaaiiGacqWFdpWCdaqhaaWcbaGaeGOmaidabaGaeGOmaidaaaaa@3087@ + {log_2_(*R*_1_/*R*_2_)}^2 ^    (4)

**Figure 9:**
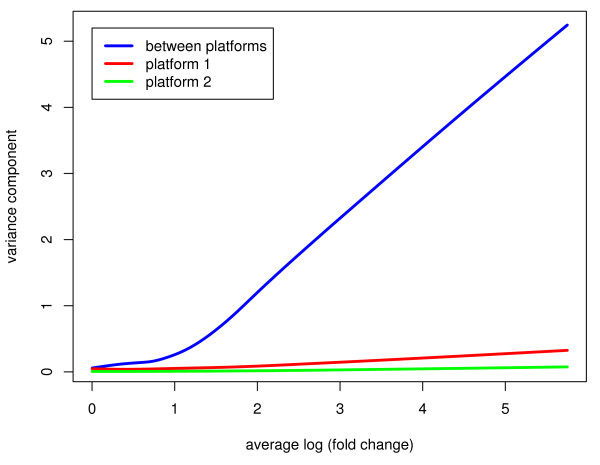
**Within- and between-platform variance components for the oligo (red) and Agilent (green) platforms, as a function of average log fold change**. The total variation between platform can be shown to be made up of the variation within each platform plus the variation between platforms which arises from different probe technologies and matching probes across technologies. It can be seen that the variance from between platform measurements is much larger than the variation within any given platform. As expected, the disagreement increases as the magnitude of the fold change increases.

The first two terms on the right-hand-side of equation 4 are the within-platform components while the third term is the between-platform component, simply the squared log-ratio of the fold-changes for this gene on the two platforms. For most genes the third term turns out to be considerably larger than the first two, particularly for genes with larger fold-changes. Figure 9 plots each of the three components of equation 4 against average log-fold change for the 3,636 matched genes when comparing the cDNA and Agilent platforms. For each component, the plot is given as a loess trend line. The between-platform component is larger than the within platform components at all fold changes but becomes dominant as the fold change increases. This relationship of within- and between-platform variation is qualitatively the same for all the combinations of platforms (data not shown). Previous work has shown that that matching genes across platforms using sequences rather than UniGene IDs reduces the differences seen between platforms [50]. It is unlikely however that the qualitative picture in which the between-platform component dominates would be materially changed.

### Comparing pre-processing methods for Affymetrix data

**Figure 10:**
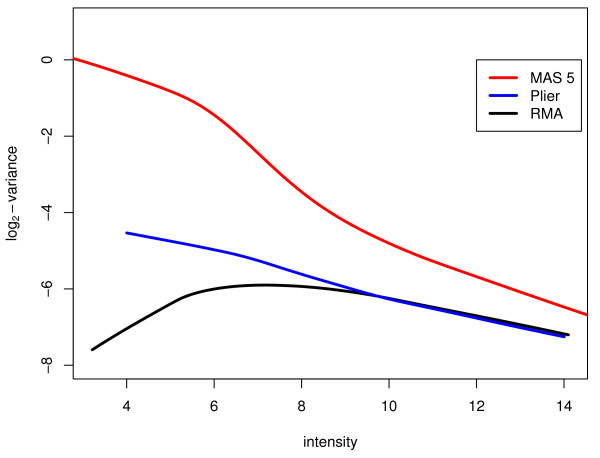
**Variance as a function of intensity for Affymetrix preprocessing algorithms**. The lowess trend curves of log_2_ϕ^
 MathType@MTEF@5@5@+=feaafiart1ev1aaatCvAUfKttLearuWrP9MDH5MBPbIqV92AaeXatLxBI9gBaebbnrfifHhDYfgasaacH8akY=wiFfYdH8Gipec8Eeeu0xXdbba9frFj0=OqFfea0dXdd9vqai=hGuQ8kuc9pgc9s8qqaq=dirpe0xb9q8qiLsFr0=vr0=vr0dc8meaabaqaciaacaGaaeqabaqabeGadaaakeaaiiGacuWFvpGAgaqcaaaa@2E8F@ versus the average log-intensity of MCF7 and Jurkat for the three pre-processing algorithms MAS5 (red), PLIER (blue) and RMA (black). It can be seen that MAS 5 gives higher variance than the other algorithms over all intensities while RMA and PLIER are almost identical at high intensities. At low intensities the RMA algorithm is more precise than PLIER, perhaps at the cost of greater bias.

All results presented above for the Affymetrix arrays use expression values for each probe-set on each array produced by the RMA software algorithm [39]. Most Affymetrix expression results published in the literature have been processed however using the Microarray Analysis Suite (MAS) software from Affymetrix, now replaced by GeneChip Operating Software [51]. Affymetrix has recently developed a new preprocessing algorithm called PLIER which incorporates some of the ideas associated with RMA. To examine how these methods might change the results, we repeated all the analysis described above using MAS and PLIER data. MAS 5.0 and PLIER were used to extract the data using Affymetrix's recommended parameters. For PLIER, quantile normalisation was used and background subtraction was selected as PM-MM with an offset of 2^4 ^= 16 added for variance stabilization at the low intensity end (Affymetrix, personal communication). The nonlinear model was fitted to the MAS 5 and PLIER data and the variances σp2
 MathType@MTEF@5@5@+=feaafiart1ev1aaatCvAUfKttLearuWrP9MDH5MBPbIqV92AaeXatLxBI9gBaebbnrfifHhDYfgasaacH8akY=wiFfYdH8Gipec8Eeeu0xXdbba9frFj0=OqFfea0dXdd9vqai=hGuQ8kuc9pgc9s8qqaq=dirpe0xb9q8qiLsFr0=vr0=vr0dc8meaabaqaciaacaGaaeqabaqabeGadaaakeaaiiGacqWFdpWCdaqhaaWcbaGaemiCaahabaGaeGOmaidaaaaa@30FE@ were recalculated. Investigation of the variances as a function of probe intensity revealed that for both MAS 5 and PLIER the variance increases at lower intensities (Figure 10). It can be seen that both RMA and PLIER give more precise results than MAS over the entire intensity range. The difference is substantial, corresponding to a four-fold increase in statistical information for most probes. For high intensities, RMA and PLIER give almost identical variance measures. At low intensities, RMA gives lower variances than PLIER. At these intensities, RMA is also returning smaller fold changes than PLIER, so it appears to be attenuating the fold changes in this intensity range in order to achieve the observed reduction in variability.

## Discussion and conclusion

This article has described a new approach to evaluate the reliability and accuracy of microarray platforms. The method consists of an easy to construct titration series and an associated statistical analysis. The global performance of each microarray platform is determined by examining the deviation of every probe on the array from its expected behavior. This differs from most previous work where the behavior of a gene is not an expected value, but rather is subject to *post hoc *validation. Implicit in much previous work is that the behavior of the whole array is inferred from a relatively small number of validated expression values.

A series of mixtures of two distinct RNA sources is used to induce a graduated series of fold changes for each probe. Our design uses one of the pure RNA samples as a common reference. This strategy ensures that the analysis can be conducted entirely in terms of log-ratios for the two-color platforms. Nonlinear regression is used to model the expected pattern by which expression must change as a function of the mixing proportions. This appears to be the first application of nonlinear regression to microarray data. The residual variation from the probe-wise regressions provides a measure of precision and the lack of fit of the curves measures deviation from response linearity for each platform. The nonlinear regression approach involves a novel concept, the idea that a consensus measure of fold change between the RNA sources can be obtained from the entire series of arrays, even though most of the arrays do not compare the pure samples. The consensus fold change provides a measure of dynamic range for each platform.

For the titration series to be most effective, the two RNA sources should contain many differentially expressed probes including a wide range of fold changes. The RNAs chosen in this article were MCF7 breast epithelial cells [52] and Jurkat T-cells [53]. Previous work involving more than 200 replicates of MCF7 versus Jurkat hybridization, as part of a large microarray quality control program, showed these lines to have a very divergent pattern of gene expression consistent with their diverse tissues of origin [54].

Four platforms were compared in this study. The ink-jet printed two-color Agilent arrays and the single channel *in situ *synthesized Affymetrix arrays gave the most consistent results with respect to the predicted behavior. Academically produced spotted cDNA and oligonucleotide arrays give somewhat more variable expression measurements, as would be expected given their lower cost and quality control. All the platforms yielded acceptable precision however. Even the least precise cDNA platform gave a typical standard deviation of only 0.23 for measured log-ratios from a single array (Table 2), which translates into variation of ± 17% for the fold changes (2^0.226 ^= 1.17). Precision is intensity dependent for the spotted arrays but far less so for the commercial arrays. The spotted arrays are virtually as precise as the more expensive platforms for high-intensity probes but the commercial platforms appear better able to resolve differential expression at lower expression levels.

The aim of this study was to evaluate the best-case scenario for each platform. In practice, the platforms may perform differently in other laboratories, particularly in less experienced hands. The hybridization process is more automated for the commercial platforms, especially Affymetrix, so these platforms can be expected to give stable results for most laboratories. Our experience in analyzing microarray data from many laboratories suggests that the academic array platforms are more subject to operator and laboratory variability and to loss of quality in inexperienced hands.

The effective precision of the two-color platforms was found to be substantially improved by allowing for probe-specific dye effects in the statistical model. This implies that microarray experiments using two-color arrays should be designed with sufficient alternation of dye assignment to allow probe-specific dye effects to be identified. The results also imply that experimenters should routinely allow for probe-specific effects in model-based analyses of two-color microarray data [46,55,56].

It was a design criterion of our study that all two-color microarrays were hybridized with identical labeled material. Each point in the titration series was labeled as a pool and then divided three ways between the platforms. This approach was taken to control for any possible titration inaccuracy or variation between probe labeling protocols. The Agilent protocol calls for labeled riboprobes generated by a proprietary kit. It is possible that our decision to standardize the labeling protocol across platforms may have contributed to the large probe-specific dye effects observed with the Agilent arrays. Nevertheless we have found in separate studies that the two protocols produce very similar results (data not shown), suggesting that any bias introduced by the labeling protocol is likely to be small.

Obtaining expression values from microarrays requires several post-hybridization data analysis steps including background correction and normalization. These steps are often collectively called *pre-processing *to distinguish them from subsequent data analysis steps. The methodology developed in this article provides a means to objectively compare different pre-processing methods for any given platform. It is our intention to make the raw expression data generated by this study publicly available. A website will be established through which researchers can interactively evaluate, for any of the four platforms, any pre-processing method which is implemented as a function in the R software environment. This is analogous to but more general than the resource provided by the affycomp software package for the Affymetrix platform [28].

It can be argued that the molecular biology literature has been slow to appreciate the extent to which the choice of pre-processing algorithm can impact on the precision of microarray data. The emphasis has often been on filtering highly variable spots rather than on ensuring low variability in the first place. This study compared three pre-processing methods for Affymetrix data and showed that the differences are substantial. The propriety Affymetrix pre-processing algorithm, the most frequently used in the literature, was found to be easily the worst of the three methods. The differences were so large that Affymetrix quantified with MAS5.0 was the worst of all the platforms considered in this study whereas Affymetrix quantified with RMA was nearly the best. The loss of information of MAS5.0 compared to RMA or PLIER varies from about 2-fold for highly expressed genes to 16-fold at low intensities. Similarly unfavorable comparisons could have been made with commonly used pre-processing methods for the two-color platforms (data not shown). This highlights the importance of using state-of-the-art pre-processing methods to get reliable data from microarray technologies.

After probe matching, the four platforms were found to broadly agree, with log-ratios across probes giving correlations of around 0.7. The differences are large enough though to establish that the platforms do differ systematically and, in fact, the majority of inter-platform disagreement is due to systematic disagreement. This agrees with qualitative observations in other studies [16,36]. A quantitative decomposition of variation into within- and between-platform components is given for the first time.

In this study, probes were matched across platforms on UniGene ID. Where there were multiple probes, or probe-sets, on a platform for the same UniGene ID, the most highly expressed probe was chosen. It is probable that better agreement could have been obtained between the platforms by BLAST searching [57] each individual probe sequence and, where there were multiple probes, attempting to match probes based on sequence similarity or on locus within a gene. Careful probe sequence matching of this type is likely to be valuable in the future for improving cross-platform agreement, but it would be unlikely to change the qualitative results presented in this study.

The presence of systematic disagreement between the platforms emphasizes the need for improved annotation and, in particular, for identification of the splice variants and other isoforms for each gene. Systematic differences have implications for how well published expression profiles for a particular phenotype will be predictive using other platforms [58]. It is widely accepted that there is a high error rate in annotation of early cDNA libraries, and despite some efforts at re-annotation, for example in the NIA 15 K mouse clone set [59], errors have tended to propagate along with the library. Recent work analyzing Affymetrix probe-sets showed a surprisingly high level of mismatches between probe sequences and the RefSeq clone sequence the probes purportedly interrogate [30], with only 62% of human U133A probe sequences being perfectly matched. The consequence of mis-annotated or mismatched probes has been shown in experiments where correlations between platforms derived from sequence verified probes are generally higher than for all probes [30]. Recent technologies such as exon and tiling arrays offer potential for elucidation of splice variants and finer annotation of microarray probes [14,60-64]. Quantifying the degree of between-platform disagreement for each gene can serve to prioritize probes for re-annotation.

## Methods

### Microarrays

Analysis was performed on four microarray platforms, two in-house and two commercial platforms. A spotted human cDNA array with approximately 10,500 probes [65] was printed at the Peter MacCallum Cancer Centre (PMCC) in Melbourne. A spotted 19.2 k 70-mer oligonucleotide array was also printed at the PMCC using oligos supplied by Compugen. Commercial microarray platforms were obtained from Agilent Technologies and Affymetrix. In situ synthesised Human 1A Oligo arrays (Agilent) printed with Agilent Sureprint Technology contain 22,000 Oligonucleotide probes. Affymetrix U133A arrays contain 17,000 in situ synthesised probe sets made using photolithographic technology.

### Cell culture and RNA extraction

MCF7, a breast epithelial cancer cell line [52], and Jurkat, a human T cell leukemia cell line [53], were selected for the experiment due to their diverse sites of origin. MCF7 cells were cultured in DMEM containing 10% FCS, and Jurkat cells were cultured in RPMI supplemented with 10 mM HEPES and 10% FCS. MCF7 cells were harvested 42 hours after addition of fresh media. Briefly, cells were first washed with 10 ml PBS prior to treatment with 3 ml trypsin for 3 minutes at 37°C. Trypsin was inactivated using 7 ml of DMEM media. Jurkat cells were collected 42 hours after adding fresh media by centrifugation. For both cell lines cell pellets were first washed with PBS before storage at -80°C. Total RNA was isolated using phenol-chloroform (TRIzol, Invitrogen) extractions and purified using column chromatography (RNeasy, Qiagen) according to manufactures instructions. RNA quality was checked by quantifying 260/280 and 260/230 ratios and gel electrophoresis (data not shown). RNA was stored at -80°C after ethanol precipitation.

### RNA titration series

A titration series was made using MCF7 and Jurkat total RNA (Table 1). To minimize variability introduced from sample preparation, master mixes from each dilution series point (A1–A6 and B) were prepared for the test samples and the reference. This master mix was used to aliquot relevant amount of RNA needed for each labeling reaction. The amount of RNA used depended on the specific protocols for the microarray platform.

### Two colour microarray platforms (Human 10.5 K cDNA, Compugen 19.2 k Human oligonucleotide arrays and Agilent Human 1A oligo array)

For Human 10.5 k and Compugen 19.2 k Human oligo arrays, 50 ug and 100 ug respectively of total RNA from test samples (A1–A6) and reference (B) were reverse transcribed using M-MLV Reverse transcriptase (Promega) incorporating AA-dUTP (Sigma). cDNA was indirectly labelled by coupling to Cy3 and Cy5 monoreactive dyes (Amersham) according to the manufacturer's protocol. To eliminate variations due to labeling, Cy3 and Cy5 labelled cDNA products belonging to the appropriate reaction number (A1–A6, and B) were pooled and proportionately divided prior to combining of the test and the reference sample for hybridisation. Repeats of each experiment were dye swapped to account for variation arising due to biased dye incorporation. After denaturation, cDNA probes were hybridised with 3.1× SSC and 50% formamide. Hybridisation was performed in a humidified HyPro_20 _System (ThermoHybaid) at 42°C for 14–16 hours. Slides were washed with 0.5 × SSC/0.01% SDS (1 minute), then 0.5 × SSC (3 minutes) and finally 0.06 × SSC (3 minutes) at room temperature. For Agilent Human 1A oligo arrays, the same indirect labelling protocol described above was used to label 50 ug of total RNA. However hybridisation and subsequent washing of the arrays were performed according to the manufacturer's protocol. Briefly, 10× control mix (Agilent) and 2× hybridisation buffer (Agilent) were added to denatured cDNA probes to. Hybridisation was performed in an oven (Robinson Scientific model 400) with rotator suited for Agilent Microarray Hybridisation chambers at 60°C for 17 hours. Slides were washed with 6 × SSC/0.005% Triton X-102 for 10 minutes at room temperature. Followed by second wash with 0.1 × SSC/0.005% Trition X-102 for 10 minutes at 4°C. All two-colour arrays were scanned using the Agilent Microarray Scanner System (G2565AA, Agilent Technologies).

### Single channel array (Affymetrix U133A GeneChip)

10 ug of total RNA from test samples (A1–A6) and reference (B) was used to prepare double strand (ds) cDNA according to manufacturer's protocol. Replicates were performed for each sample. Biotin labelled cRNA was synthesized from ds cDNA using ENZO BioArray High Yield RNA transcript kit (Affymetrix). cRNA from appropriate reactions was pooled and divided before fragmentation. 30 ug of pooled cRNA was fragmented according to manufacturer's instructions. Fragmented cRNA was then split into two 15 ug aliquots, which was used to make hybridization solution. 2/3 of the hybridization mix, 10 ug of cRNA, was hybridized at 45°C and 60 RPM for 16 hours in an oven with a rotor (Hybridization oven 640, Affymetrix). Hybridized cRNA was then washed and stained according to antibody amplification stain protocol for Eukaryotic RNA. Slides were first washed with non-stringent wash buffer (6× SSPE and 0.01% Tween-20) at 30°C followed by stringent wash buffer (100 mM MES, 0.1 M [NaH], 0.01% Tween-20) at 50°C. GeneChips were stained with SAPE solution at 35°C prior to washing with non-stringent wash buffer. GeneChips were then stained with biotynlated anti-SAPE antibody at 35°C. Subsequent staining with a second SAPE solution for biotynlated anti-SAPE was followed by final wash with non-stringent wash buffer. Chips were then scanned twice using 2500 Scanner. Data was extracted using Affymetrix GeneChip^® ^Operating Software (GCOS) Version 1.0.

### Preprocessing and normalization

Expression data from the three two-color platforms was pre-processed in the same way using methods which have been shown to reduce variability [47]. Intensity data was extracted from the red and green scanned images using the SPOT software package [66]. The intensities were corrected for background luminescence by subtracting the "morph" background estimate [37] giving background corrected red (R) and green (G) intensities for each spot on the arrays. These were then summarized into log-ratios M = log_2_R/G and average log-intensities A = (log_2_R+log_2_G)/2 for each spot. The log-ratios were normalized for each array using print-tip loess normalization [38] implemented in the limma software package [56] for R [67]. The Agilent ink jet technology doesn't use print tips so global loess normalization was used for the Agilent arrays.

Affymetrix arrays have a qualitatively different design than the other platforms. Not only are they single instead of two-channel but they use multiple probes, a "probe set", to represent each gene or EST. The platform also contains mismatch probes designed to test non-specific hybridization. Pre-processing methods for this platform must therefore be different from the two-color platforms. We extracted summary log-expression values for each probe set on each array using the robust multi-array average (RMA) algorithm, implemented in the affy software package for R, which has been shown to substantially reduce variability especially for less highly expressed genes [39]. For comparison purposes, summary expression values were also obtained using Affymetrix's proprietary pre-processing software products MAS 5.0 and PLIER. In this article, the term "probe" for the Affymetrix platform will refer to a probe set.

In each case, pre-processing was applied to all probes on the arrays, including control probes. The only exception were blanks (ControlType = ignore) on the Agilent arrays, which were removed prior to normalization. For all platforms, control probes were removed prior to the analyses described in RESULTS.

All arrays and hybridizations were examined by standard quality assessment procedures [68] and were found to be of excellent consistent quality.

### Nonlinear regression

The main evaluation method developed in this paper is based on nonlinear regression models which relate the mixing proportions in the titration series to the expression log-ratios, for the two-color platforms, and to the log-expression values, for the Affymetrix platform. The unknown parameters in the models, the actual expression levels for each probe, were estimated for each probe and for each platform using the nonlinear least squares (nls) function in the R programming environment [69].

## Authors' contributions

AJH conceived the experiment, oversaw the hybridizations and drafted the experimental parts of the manuscript. AO undertook and helped develop the statistical analyses, and drafted the analysis parts of manuscript. DSD carried out the hybridizations. DDLB advised on the interpretation of the results and helped draft the manuscript. GKS helped design the experiment, conceived the overall strategy of the statistical analysis, and finalized the manuscript. All authors read and approved the final manuscript.
